# An Expanded Multi-Organ Disease Phenotype Associated with Mutations in *YARS*

**DOI:** 10.3390/genes8120381

**Published:** 2017-12-11

**Authors:** Anna Tracewska-Siemiątkowska, Lonneke Haer-Wigman, Danielle G. M. Bosch, Deborah Nickerson, Michael J. Bamshad, University of Washington Center for Mendelian Genomics, Maartje van de Vorst, Nanna Dahl Rendtorff, Claes Möller, Ulrika Kjellström, Sten Andréasson, Frans P. M. Cremers, Lisbeth Tranebjærg

**Affiliations:** 1 DNA Analysis Laboratory, Wrocław Research Centre EIT+, 54-066 Wrocław, Poland; 2 Department of Human Genetics, Radboud University Medical Center, 6525 GA Nijmegen, The Netherlands; Lonneke.Haer-Wigman@radboudumc.nl (L.H.-W.); D.G.M.Bosch-2@umcutrecht.nl (D.G.M.B.); Maartje.vandeVorst@radboudumc.nl (M.v.d.V.); Frans.Cremers@radboudumc.nl (F.P.M.C.); 3 Bartiméus, Institute for the Visually Impaired, 3700 BA Zeist, The Netherlands; 4 Donders Institute for Brain, Cognition and Behavior, Radboud University Medical Center, 6525 EN Nijmegen, The Netherlands; 5 Department of Genome Sciences, University of Washington, Seattle, WA 98195, USA; debnick@uw.edu; 6 Department of Pediatrics, University of Washington, Seattle, WA 98195, USA; mbamshad@uw.edu; 7 University of Washington, Seattle, WA 98195, USA; 8 Department of Clinical Genetics, The Kennedy Centre/Rigshospitalet/, DK-2600 Glostrup, Denmark; nanna.dahl.rendtorff@regionh.dk (N.D.R.); tranebjaerg@sund.ku.dk (L.T.); 9 Audiological Research Centre/Swedish Institute of Disability Research, University Hospital Örebro, Örebro University, 701 85 Örebro, Sweden; claes.moller@oru.se; 10 Department of Clinical Science Lund, Ophthalmology, University of Lund, 221 00 Lund, Sweden; ulrika.kjellstrom@med.lu.se (U.K.); sten.andreasson@med.lu.se (S.A.); 11 Institute of Clinical Medicine, University of Copenhagen, DK-2200 Copenhagen N, Denmark

**Keywords:** *YARS*, syndromic retinitis pigmentosa, whole exome sequencing

## Abstract

Whole exome sequence analysis was performed in a Swedish mother–father-affected proband trio with a phenotype characterized by progressive retinal degeneration with congenital nystagmus, profound congenital hearing impairment, primary amenorrhea, agenesis of the corpus callosum, and liver disease. A homozygous variant c.806T > C, p.(F269S) in the tyrosyl-tRNA synthetase gene (*YARS*) was the only identified candidate variant consistent with autosomal recessive inheritance. Mutations in *YARS* have previously been associated with both autosomal dominant Charcot-Marie-Tooth syndrome and a recently reported autosomal recessive multiorgan disease. Herein, we propose that mutations in *YARS* underlie another clinical phenotype adding a second variant of the disease, including retinitis pigmentosa and deafness, to the spectrum of *YARS*-associated disorders.

## 1. Introduction

Aminoacyl transfer RNA synthetases (ARSs) are a group of enzymes catalyzing linkage of transfer ribonucleic acid (tRNA) to amino acids. In principle, these proteins are thought to ‘read the genetic code’. Aminoacyl-tRNA, which is created in this process, enables incorporation of amino acids into a growing polypeptide chain at a position determined by the anticodon loop sequence of the specific tRNA. Each of the synthetases is highly specific and has an error rate of around 10^−4^–10^−5^, which is provided by their proofreading activity [1]. Since aminoacylation is a process that runs in both the cytoplasm and mitochondria, each of the ARS has its mitochondrial counterpart.

Hitherto, many mutations in ARSs were linked to genetic diseases. Not all of them are directly affecting the synthetase activity; hence, they are believed to act also outside of this function. For instance, some may be acting through the immune system or angiogenesis stimulation. Additionally, they may also form complexes with other proteins, influencing various pathways [2].

The spectrum of disease phenotypes associated with mutations in ARSs is very wide. In general, disorders caused by heterozygous mutations, exerting either haploinsufficiency or a dominant negative effect (or a combination of both), display later onset and affect mostly the peripheral nervous system. In contrast, homozygous or compound heterozygous mutations mostly result in early-onset, severe phenotypes, affecting multiple tissues [3].

Heterozygous mutations in *YARS*, an *ARS* gene which encodes for tyrosyl-tRNA synthetase, were first identified by Jordanova et al. [4] in dominant intermediate Charcot-Marie-Tooth (DI-CMT) disease (OMIM # 118220), which is associated with motor and sensory neuropathy. Lately, a novel phenotype resulting from bi-allelic *YARS* mutations was described by Nowaczyk et al. [5]. In contrast to DI-CMT, this disorder shows no evidence of motor or sensory demyelinating or axonal neuropathy. It is characterized by failure to thrive, developmental delay, and abnormalities in multiple organs. The siblings harboring these mutations had distinct facial features and thinning of the corpus callosum, fatty liver, hypotonia, and joint hypermobility (Table 1).

Here, we present a case with homozygous *YARS* mutation and discuss similarities and differences between our patient and other individuals bearing *YARS* (and other *ARS*) mutations described in literature.

### Clinical Case Report

The affected individual was a female, 26 years old at the time of analysis. She was born at term (birth weight 2455 g, length 47 cm) after an uncomplicated delivery. The proband had unstable blood sugar levels with episodes of hypoglycemia in the neonatal period.

In the first year of life, the patient had a high level of blood platelets and signs of hyperactive bone marrow on bone marrow biopsy, without suspicion of malignancy. She also had cholestasis and prolonged jaundice up to six months of age. A sweat test was normal.

The patient’s parents were of European origin, healthy and not known to be closely related. The grandparents were born in two small villages (within 16 km distance) in Northern Sweden, hence the parents were suspected to be cryptically related. The proband had an unaffected sister, who was born eight years later.

The proband had no facial dysmorphic features. At three to four months of age, she presented with nystagmus and profound hearing impairment, which was suspected to be congenital. At age four months, she was hospitalized because of poor weight gain. She was tube fed for an extended period of time due to poor weight gain. In the first year of life, she experienced episodes of hypoglycemia and a liver biopsy was performed. The biopsy revealed fatty liver; however, the liver function as evaluated by biochemical parameters underwent subsequent stabilization and she showed no clinical insufficiency later in life. Liver biopsy was repeated at six years and showed only minor fibrotic changes. She was treated with hearing aids, but at 21 years of age she communicated using sign language. Cochlear implantation was refused.

**Figure 1:**
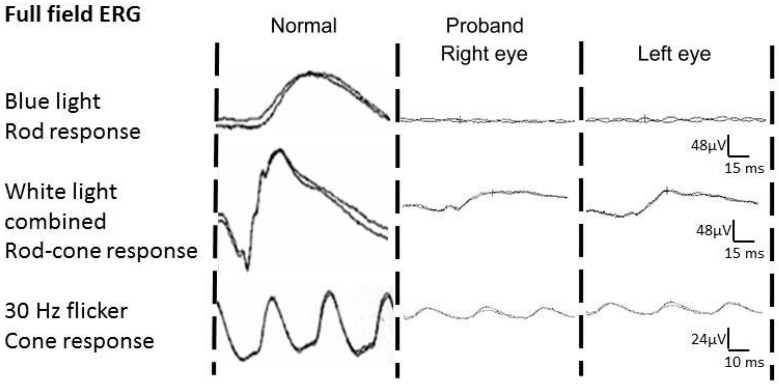
Patient’s electroretinogram showing no residual rod responses and decreased, but still detectable cone responses in both eyes. ERG: electroretinography.

Full field electroretinography (ffERG) was recorded during general anesthesia mainly according to the standardized protocol for clinical electroretinography (ISCEV) using a Nicolet analysis system (Nicolet Biomedical Instruments, Madison, Wisconsin) at the age of eight years. These recordings confirmed a rod-cone degeneration with no residual rod responses but still remaining cone function. Delayed cone response also confirmed progressive rod-cone degeneration (Figure 1). Fundus examination revealed pigmentation, degeneration, and atrophy in the macular area, but not with the typical pattern observed in Usher syndrome. The proband is considered deaf with severe visual handicaps including no night vision, problems with glare, reduced visual field, and low central vision. 

Psychomotor development was normal. The proband did not display intellectual disability and walked without support at age 13 months, but had poor balance. A computed tomography (CT) scan, performed at age six months, showed agenesis of corpus callosum. At two to three years of age she had episodes of generalized seizures. The patient displayed primary amenorrhea. During the first year of life, hypotonia was pronounced, electromyogram showed low amplitudes, and was suspected of indicating myopathy. Therefore, muscle biopsy was performed, revealing disputable minor abnormalities. However, there were no clear signs of either mitochondrial or myopathic abnormalities, and subsequent electron microscopy returned normal results. Plasma lactate was elevated.

## 2. Materials and Methods

### 2.1. Genetic Analyses

Informed consent was obtained from the patient and participating relatives. This study adhered to the tenets of the Declaration of Helsinki and was approved by local bioethics committee. Study of genetic causes of deafness with associated clinical abnormalities was approved by a Regional Research Ethical Committee in Denmark with the file number (KF) 01-234/02 and (KF) 01-108/03.

Genomic DNA of participating individuals was isolated from peripheral blood using standard methods. A number of clinical genetic tests preceded exome sequencing.

Since the patient had congenital nystagmus and degeneration of the retina, she was initially tested for variants associated with Leber congenital amaurosis (LCA) using arrayed primer extension (APEX) chip technology, with 451 variants in 11 genes (Asper Biotech, Turku, Estonia). Subsequently, all exons and intron-exon boundaries of *CEP290* (including the c.2991 + 1655A > G mutation) were Sanger sequenced. Because of her deafness, the protein-coding exon of *GJB2* was sequenced. Since Usher syndrome was in the differential diagnosis, she was also tested using APEX, with 612 known variants in nine known Usher syndrome-associated genes: *ADGRV1*, *CDH23*, *CLRN1*, *DFNB31*, *MYO7A*, *PCDH15*, *USH1C*, *USH1G*, and *USH2A.*

Genome-wide homozygosity mapping was performed using Affymetrix 5.0 SNP array (Affymetrix, Santa Clara, CA, USA) containing approximately 500,000 SNPs. Three genes (i.e., *IMPDH1*, *WFS1*, and *PEX1*) suspected of being (partially) responsible for the phenotype, that were found in the largest homozygous regions, were screened via Sanger sequencing (Supplementary Table S1).

### 2.2. Exome Report

Library construction and exome capture have been automated (Perkin-Elmer Janus II) and libraries underwent exome capture using the Roche/Nimblegen SeqCap EZ v2.0 (~36.5 MB target). To facilitate optimal loading, all samples were sequenced on the Illumina MiSeq platform prior to deep sequencing. Barcoded exome libraries were hand pooled prior to clustering (Illumina cBot) and loading. Massively parallel sequencing-by-synthesis with fluorescently labeled, reversibly terminating nucleotides was carried out on the HiSeq sequencer.

Data Quality Control (QC) included an assessment of: (1) total reads (minimum of 50 million PE50 reads); (2) library complexity; (3) capture efficiency; (4) coverage distribution: 90% at 8X read depth required for completion; (5) capture uniformity; (6) raw error rates; (7) transition/transversion ratio (Ti/Tv); (8) distribution of known and novel variants relative to The Single Nucleotide Polymorphism database (dbSNP); typically <7%; (9) fingerprint concordance >99%; (10) sample homozygosity and heterozygosity; and (11) sample contamination validation. Exome completion was defined as having ≥90% of the exome target at ≥8× coverage and >80% of the exome target at ≥20× coverage. Typically, this requires mean coverage of the target at 50–60×. An automated pipeline for annotation of variants, the SeattleSeq Annotation Server (http://gvs.gs.washington.edu/SeattleSeqAnnotation/), was used.

To find the de novo variants, a trio analysis was performed. For each sample, the variants of the patient were pre-filtered and high quality rare coding variants remained. The remaining variants that were also present in the parental Variant Call Format (VCF) files were annotated with paternal, maternal or shared, and for the other variants variant calling (mpileups) were performed on the parental binary SAM—sequence alignment data files (BAM files) using SAMtools (version 0.1.9). In case the variant was seen in more than 1% of the parental reads, the variants were also annotated as paternal, maternal or shared, the other variants were annotated as possible de novo.

The number of variants obtained for each exome of the trio was 26,493 (mother, I:2), 25,415 (father, I:1) and 25,741 (proband, II:1). The average read depth of target bases was 56× (M) 54× (F) and 57X (P), respectively, with 91% of target bases being covered ≥10×.

Only variants located in exons and canonical splice site regions were considered. Only those with quality-by-depth above 500 displaying at least ≥20% variant reads for each position were used (24,701 variants). The frequency cutoff for in-house Nijmegen database of ~800 exomes, as well as other projects (dbSNP 139, Exome Sequencing Project (ESP), Genome of the Netherlands (GoNL) and Wellderly) was set to 0.5%. This filtering resulted in 547 variants. These contained 244 missense variants which were assessed for pathogenicity clues using combined annotation dependent depletion (CADD) [6] scores and only those with a score above 15 were considered (76 variants). Additionally, there were 86 splice site variants (7 covering canonical splice sites), 33 frameshift and 21 in-frame insertions/deletions, and 7 nonsense mutations. Synonymous variants (134), were omitted in the analysis if they were not predicted to have an effect on a splice site. Integrative Genomics Viewer v. 2.3.14 was used to visualize the BAM data [7], and AlaMut Visual software 2.6 (Interactive Biosoftware, Rouen, France) showed nucleotide and amino acid conservation scores.

## 3. Results and Discussion

We report a distinct phenotype, which is hypothetically caused by a homozygous variant in *YARS*. The condition is characterized by rapid retinal degeneration, profound hearing impairment, agenesis of corpus callosum, primary amenorrhea, and liver affection (fatty liver and subsequent mild fibrotic changes) in a patient of Swedish origin.

Screening for known mutations performed with Asper chips for retinitis pigmentosa (RP) and Usher syndrome, as well as GJB2 sequencing for deafness, showed no causative variants. Genome-wide homozygosity mapping revealed multiple large homozygous regions, indicating probable parental relatedness in the family. There were nine homozygous regions ≥3 Mb, which consisted altogether of 84 Mb, suggesting that the parents might be related in the second degree [8] (Supplementary Table S1). However, sequencing of the three suspected genes residing in the first, third, and fifth largest homozygous regions (*IMPDH1*, *WFS1*, and *PEX1*) did not yield a causal variant.

As this was a simplex family with healthy parents, the phenotype of the proband could have been inherited in either an autosomal recessive or de novo dominant pattern; however, after trio analysis, the latter possibility was less likely. Identified de novo mutations appeared to be either benign, artificially introduced or were false positives of maternal or paternal origin where parental sample did not display sufficient read depth and the variant was not called. Therefore, we prioritized discovered candidate genes using recessive model of inheritance. Candidates found under an X-linked model were considered less likely because of the female sex of the patient.

For the most stringent, initial filtering, besides considering only rare variants, only non-synonymous and canonical splice site alterations were considered (*n* = 156). Seven genes bore multiple (2–7) heterozygous variants, and 22 variants were homozygous (>90%). These were mostly indels, which upon closer examination frequently turned out to be false-positives.

After detailed analysis of the candidate variants, there were two alterations matching the presumed inheritance pattern that were not false positives. These were two homozygous variants in *CELA1* and *YARS* (Supplementary Table S2).

A homozygous alteration in *CELA1* was reported, which was a single large deletion leading to a frameshift. *CELA1*, coding for chymotrypsin-like elastase family, member 1, is involved in digestion of elastin present in elastic fibers, as well as other proteins, such as hemoglobin [9]. It was excluded as there is another, very common frameshift mutation present in a homozygous state in healthy individuals resulting in premature termination of the protein [10], which would suggest the redundancy of the enzyme.

**Figure 2:**
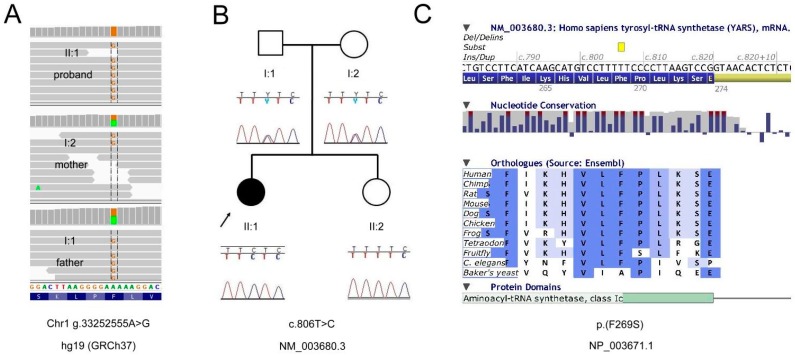
Pedigree and mutation data for the *YARS* gene. (**A**) binary SAM (BAM) file visualization in Integrative Genomics Viewer (IGV) software [11] displaying the variant site in the whole trio; (**B**) Familial segregation analysis with Sanger sequencing; (**C**) AlaMut Visual amino acid conservation in animal orthologs.

In *YARS*, we discovered a homozygous missense variant, c.806T > C, (p.(F269S), NM_003680), which was heterozygously present in the healthy parents and absent in the healthy sister (Figure 2A,B). YARS is responsible for tyrosyl-tRNA aminoacylation. It also plays a secondary role in cellular apoptosis, angiogenesis, and immune response. The CADD score, which is a widely-used pathogenicity assessment tool incorporating many prediction algorithms [6], for this substitution was relatively high, 20.5. Nucleotide conservation in this locus was also high (PhyloP 4.8). Phenylalanine residue was conserved up to nematodes (*C. elegans*) (Figure 2C). The physico-chemical properties of phenylalanine and serine differ significantly (Grantham score of 155).

Previous reports for mutations in *YARS* described missense mutations causing DI-CMT disease, a neurological disorder (OMIM # 118220). However, CMT caused by *YARS* variants is inherited in autosomal dominant patterns, whereas our findings suggest our patient’s novel phenotype was inherited in an autosomal recessive pattern. Nowaczyk et al. recently published a novel *YARS*-associated phenotype that partially overlaps the phenotype of the patient we describe [5]. They reported two siblings with compound heterozygous *YARS* mutations with a condition affecting the liver, muscle, and brain (Table 1). Our proband was similarly diagnosed with fatty liver disease verified by biopsy, but it subsequently stabilized and no clinical liver insufficiency was noted later in her life. In contrast with the Nowaczyk et al. study, she did not have any specific facial features.

Siblings reported by Nowaczyk et al. had thinning of corpus callosum whereas agenesis of corpus callosum was reported in our patient. Hypotonia was observed in all three individuals. Neither sibling described by Nowaczyk et al. was reported to have retinal or vestibular abnormalities.

The spectrum of disease phenotypes associated with mutations in the mitochondrial aminoacyl transfer RNA synthetases genes is expanding with new features. Very recently, in a child with optic atrophy, retinal bone spicule pigmentation, absent patella reflexes and multiple cerebellar and supratentorial white matter multifocal changes as well as demyelinating polyneuropathy, Peragallo et al. [12] discovered association with missense mutations in the *AARS2* gene.

In their recent review, Meyer-Schuman and Antonellis proposed mechanisms which could underlie the similarities and differences between distinct *ARS*-mutation-driven phenotypes [3]. The authors point out that, hitherto, mutations in 31 *ARS* genes, both mitochondrial and cytoplasmic, have been associated with recessive disorders. All of the involved tissues require high amounts of energy; therefore the central nervous system is particularly affected, even in case of cytoplasmic ARSs. There is a wide spectrum of phenotypic manifestations, including all symptoms described in our patient. Some of them are overlapping, present in many syndromes (such as liver disease, ovarian failure, or hypotonia). However, there are also mutations in genes that affect specific tissues, such as retina and inner ear, causing Usher syndrome. Puffenberger et al. have reported a mutation in another tRNA synthetase gene—histidyl-tRNA synthetase (*HARS*) in Usher syndrome type IIIB [13]. Deafness is also present in diseases caused by alterations in other *ARS* genes. Alterations in other *ARS* genes, both mitochondrial and cytoplasmic, have been known to cause syndromes involving RP and/or deafness (*EARS2*, *HARS2*, *FARS2*, and *SARS2*) [13,14,15,16,17] (reviewed in Yao and Fox 2013 [18]). It is noteworthy that mutations in HARS2 are associated with a disorder called Perrault syndrome 2 [15] (OMIM # 614926), characterized by congenital severe hearing impairment and dysgenesis of the ovaries in females. Our patient had primary amenorrhea resulting from ovarian dysgenesis associated with *YARS* mutations, which has never been reported (Table 1). The female with other *YARS* mutations reported by Nowaczyk et al. was too young to demonstrate such features. 

Dominant disorders associated with *ARS* mutations encompass Charcot-Marie-Tooth syndrome and distal hereditary motor neuropathy. This entity is resembling CMT disease in terms of distal limb muscle atrophy; however, the affected individuals do not display sensory involvement. In many cases, parents of individuals suffering from recessive *ARS*-mediated disorders did not display any symptoms. Our patient’s parents were healthy and did not display any neuropathy features. In animal models, ARSs null alleles are lethal, which is corroborating the fact that in humans recessive genotypes consist of milder mutations—either missense on both alleles or one null allele and one missense mutation. Patients with dominant inheritance pattern usually harbor missense mutations (one in-frame deletion was reported). Nevertheless, it is possible that certain mutations are causing the dominant form, and others do not. Incomplete penetrance and late onset of dominant *ARS*-associated phenotypes makes interpreting these connections still challenging.

**Figure 3:**
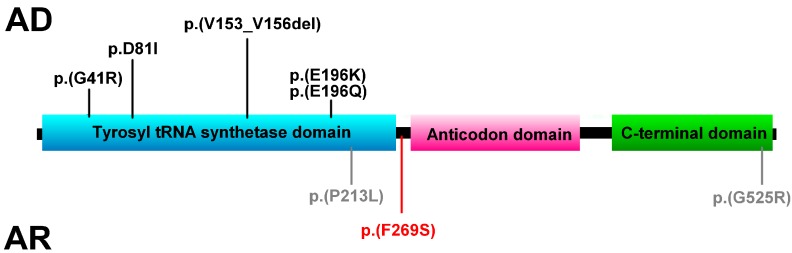
*YARS* domains and locations of autosomal dominant (AD) mutations causing CMT (black) and autosomal recessive (AR) mutations (grey—Nowaczyk et al.; red—this study).

Mutations causing CMT are all located in catalytic domain of YARS (amino acids 20 to 256). The mutations in *YARS* causing the phenotype reported by Nowaczyk et al. are located near the end of this domain (p.(P213L)) and in the C-terminus (p.(G525R)) (Figure 3). The mutation we report p.(F269S) is located just outside the tyrosine tRNA ligase domain, hence it may exert a completely different impact on the protein and cause additional phenotypic features.

The p.(Y454S) mutation causing Usher syndrome in *HARS* affects an amino acid residue located in the interface between the catalytic domain and anticodon binding domain. The altered residue reduces the maximal forward reaction velocity nearly two-fold [13]. Because our mutation affects a residue in a homologous region of the protein, it may also exert a comparable effect.

The effect of these mutations on tRNA charging has been confirmed in many different functional assays. Loss-of-function therefore would be the molecular mechanism of the disease; as for the dominant mutations, the mechanism is not clear. Currently, there are two hypotheses as to why certain *ARS* mutations cause tissue-specific phenotype despite the fact that protein translation is crucial to all tissues. These mutations might abolish expression of cell type-specific proteins acting in a codon-dependent manner. Another possibility is that specific tRNA expression (variable for different tissues) may modify the cell-specific consequences of this deficiency [3]. Further studies are required to understand the mechanisms underlying these differences.

Our findings seem to be compatible with the fact that all of the other CMT genes are associated with a distinct phenotype inherited in an autosomal recessive manner (*AARS*, *GARS*, *HARS*, *MARS*, and *YARS*) [5,13,19,20,21], and they are highly suggestive of an expanded phenotype which may be attributed to mutations in *YARS*. The clinical picture of the patient studied does not resemble CMT or the disease discovered by Nowaczyk et al.; rather, it may be an Usher-like or Perrault-like syndrome with additional features. Nevertheless, further analyses, such as genetic studies of another family with same phenotype, animal models, or functional in vitro tests are required to confirm our hypothesis.

## Figures and Tables

**Table 1:** Phenotype comparison between the patient described in this study and other patients with variants in *YARS* or *HARS2*.

	This Study	Nowaczyk et al.		DI-CMT Syndrome	Perrault Syndrome 2
**Patient no.**	II:1	Sibling 1	Sibling 2	Multiple patients	Multiple patients
**Gender**	F	M	F	F/M	F
**Mutations**	*YARS* p.(F269S)/p.(F269S)	*YARS* p.(P213L)/p.(G525R)	*YARS* p.(P213L)/p.(G525R)	*YARS* p.(G41R)/+ p.(E196K)/+ p.(D81I)/+ p.(V153_V156del)/+	*HARS2 (Many)*
**Abnormality of corpus callosum**	+++ (Agenesis)	+ (Thinning)	-	- (Thinning observed in other forms of CMT)	-
**Fatty liver**	+ (Transient)	++	++	-	-
**Specific facial features**	-	+	+	-	-
**Hypotonia**	+	+	+	+	-
**Retnititis pigmentosa**	+	-	-	-	+
**Deafness**	+	-	-	-	+
**Primary amenorrhea**	+	n.a.	n.d.	-	+

DI-CMT: dominant intermediate Charcot-Marie-Tooth disease; n.a.: not available, n.d.: not determined.

## Supplementary Materials

The following are available online at www.mdpi.com/2073-4425/8/12/381/s1. Table S1: Candidate genes within patients’ homozygous regions, Table S2: Candidate variants derived from exome sequencing.
